# Visible name changes promote inequity for transgender researchers

**DOI:** 10.1371/journal.pbio.3001104

**Published:** 2021-03-09

**Authors:** Leo Chan Gaskins, Craig R. McClain

**Affiliations:** 1 Duke University Marine Lab, Beaufort, North Carolina, United States of America; 2 Louisiana Universities Marine Consortium, Chauvin, Louisiana, United States of America

## Abstract

Academic journals allowing for invisible name changes is a matter of fundamental respect and dignity for trans researchers, but there are currently no acceptable choices when dealing with name changes in a publishing record. This Perspective article proposes a centralized name change solution through ORCID iD to relieve the burden on trans researchers to contact every journal they have previously published with.

## Teaser

Allowing for invisible name changes is a matter of dignity for trans researchers. This would prevent their own publication record from outing them without their consent. A single, centralized name change request through ORCID iD would alleviate the burden of changing each publication individually.

LGBTQ+ communities are a marginalized and disenfranchised group in science. In academia, the representation of LGBTQ people is 17% to 21% less than expected [1,2]. These communities often face more difficult career experiences than their non-LGBTQ counterparts [3]. Over 30% of those LGBT scientists surveyed described their departments as “uncomfortable,” 40% agreed with the statement “employees are expected to not act too gay,” 20% experienced and 40% observed exclusionary behavior, and 33% considered leaving their workplace in the last year [3]. This exclusionary behavior permeates science, and studies show that this decreases retention of LGBQ science, technology, engineering, and mathematics (STEM) faculty members [4]. Improvement is clearly needed in the policies and culture of academia as related to LGBTQ+ communities.

Transgender academics may also face unique barriers. Among trans scientists in physics, approximately 20% did not have a bathroom they felt safe using, only approximately 60% had colleagues that used their preferred pronouns, only approximately 50% had their healthcare needs covered, and 60% observed and 49% directly experienced harassment [3]. Reflecting a need to avoid unsafe work environments, more than 40% of academics identifying as LGBTQ have not come out to colleagues [5]. If outed to their employers, 47% faced consequences such as being fired, losing a prospective job, or not being promoted [6]. Even if they retain their job, 90% reported being harassed or discriminated against in the workplace [6].

Respecting a transgender person’s chosen name and pronouns is a matter of dignity and safety [7]. Hearing others call them by their chosen name and pronouns is an important form of acknowledgement and respect; this affirmation can strongly reduce negative mental health outcomes [8]. Their former name given at birth, known as a dead name, can be associated with trauma [9] including harassment, violence, abuse, physical and sexual assault, as well as a gender they do not identify with. The continued reference to a dead name or misgendering is an indignity that can resurrect past trauma and expose trans people to future harassment and violence [9]. We argue that the safety and intellectual freedom that are fundamental values of academia are antithetical to misgendering or dead naming transgender colleagues. Yet, this policy is embedded into how transgender academics are forced to interact with their publishing records.

This ability to protect and control personal information as a transgender academic is vital to the safety, longevity, and growth of their careers. Yet, publication records create a major liability for trans researchers. An academic that comes out as transgender and changes their name after having published using their dead name faces only 3 options that are equally unacceptable.

One, a transgender academic may choose to leave the name unchanged. This creates a public record on the curriculum vitae, the individual publications, and online indexing sites (e.g., ORCID, Google Scholar, and ResearchGate) that allows for discrimination during interviews, the tenure process, and from colleagues [10]. Moreover, this leaves their dead name on record causing further trauma for transgender academics.

Two, a transgender academic may choose to not acknowledge previous work. In a field where publications are the currency of a life’s work, this would not only damage career prospects but also cause others to undervalue a researcher’s contributions in the field. Even this extreme option creates a publication gap that raises further questions and potentially leads to outing.

Three, a transgender academic may choose to request a name change with the separate journals they have published in with their former name. Presently, most academic journal name change policies involve issuing a correction, which draws attention to the former name and gender change. Moreover, journal policies vary and require a burden on the transgender academic to navigate this process, which could potentially involve several journals. In all these cases, by leaving the name unchanged on older publications or making a correction with a journal, attention is drawn to the dead name, increasing the potential for discrimination and harassment. There is currently no systematic process to do this invisibly.

We advocate for a systematic process for invisible name changes. While we appreciate the concept of the “permanence of the scholarly record,” in order to create an ethical publishing space that is safe for all individuals, academic journals should not act as gatekeepers for a transgender person’s ability to protect and control their own information and narrative (in addition to the safety issues associated with this information). Allowing non-visible name change policies returns that control to the individual. Additionally, it can lessen the potential for discrimination against transgender individuals and promote the equality and advancement of transgender researchers across academia.

We are not the only people advocating for this policy change, and there is consensus and support being built for invisible name changes, with some publishers incorporating this policy already, including PLOS, Association of Computing Machinery (ACM), American Chemical Society (ACS), American Psychological Association (APA), Wellcome Open Research, Cell Press, Wiley, and American Geophysical Union (AGU). We applaud these early adopters, but many journals retain outdated policies and are silent on transgender issues. In addition, while journals can easily adopt individual processes to allow for invisible name changes, which we support, this still places considerable burden on an academic to work through these varying processes at each journal. We advocate for a centralized process that lessens this burden.

ORCID currently serves as a mechanism to provide a persistent digital identifier, free of gender. ORCID iD is under the control of the individual researcher, and thus, ORCID could also serve as a single mechanism to request name changes. Currently, ORCID is designed to pull information from journals. But it could be further developed to allow information to be pushed in both directions so that a single name change request could be issued to ORCID, which would then notify appropriate journals and referencing services. We envision that journals would have these requests undergo human review to avoid situations of potential misuse. This proposed mechanism reduces the burden on academics who may need to request names changes across multiple journals.

Any process should not require legal name or gender change documents. This is not required of cisgender academics, e.g., marital name changes, nor should it be required of transgender academics. The legal name change process creates financial and discriminatory barriers within academia [10]. If it is even an option, many countries require surgery for gender marker changes, not typically covered by insurance. In the United States, more than 55% of transgender individuals who sought coverage for transition-related surgery and 25% of those who sought coverage for hormones were denied [7]. The name and gender change process itself, not even existing in some countries, also requires fees, and the cost presents a prohibitive and major barrier [7].

The invisible name change process we advocate for benefits communities well beyond the transgender community. Changes in name due to marriage indicate marital status. In the US, more than half of states prohibit discrimination based on an employee’s or applicant’s marital status. In academia, hiring committees have been shown to consider a woman’s marital status when selecting hires [11]. Moreover, previous names may be associated with trauma due to domestic violence and abuse. Separation from the previous name represents a significant step in the healing process. Also importantly, an invisible name change allows women fleeing domestic violence greater protection and confidentiality [12,13].

While we identify 3 mechanisms currently available to transgender academics, a fourth does exist. Out yourself in a Perspective article so that your publication record no longer outs you. While in part we intend this statement flippantly, it does highlight that academia requires career risks of marginalized groups to both highlight issues and instigate change. An additional performance of their trauma is required in public forums as exemplars for change. Here, we use one of our own sets of experiences to highlight a substantial change needed in academia. Journals allowing invisible name changes would promote equity in science for transgender researchers and would be an important step forward. The ability to control their personal information is a fundamental right. But to truly make academia a friendly place for trans people, we cannot expect them to thrive in an unfriendly environment. We must work together to alter the system itself so that it truly welcomes, listens to, and values diverse voices, and provides them with equal opportunities.
